# Credit where credit is due: Pakistan’s role in reducing the global burden of reproductive, maternal, newborn, and child health (RMNCH)

**DOI:** 10.1186/s12961-015-0035-6

**Published:** 2015-11-25

**Authors:** Abdul Ghaffar, Shamim Qazi, Iqbal Shah

**Affiliations:** Alliance for Health Policy and Systems Research, World Health Organization, 20 Avenue Appia, Geneva, Switzerland; World Health Organization, Geneva, Switzerland; Harvard T.H. Chan School of Public Health, Boston, MA USA

**Keywords:** Capacities, Context, Global knowledge, Incentives

## Abstract

Factors contributing to Pakistan’s poor progress in reducing reproductive, maternal, newborn, and child health (RMNCH) include its low level of female literacy, gender inequity, political challenges, and extremism along with its associated relentless violence; further, less than 1% of Pakistan’s GDP is allocated to the health sector. However, despite these disadvantages, Pakistani researchers have been able to achieve positive contributions towards RMNCH-related global knowledge and evidence base, in some cases leading to the formulation of WHO guidelines, for which they should feel proud. Nevertheless, in order to improve the health of its own women and children, greater investments in human and health resources are required to facilitate the generation and use of policy-relevant knowledge. To accomplish this, fair incentives for research production need to be introduced, policy and decision-makers’ capacity to demand and use evidence needs to be increased, and strong support from development partners and the global health community must be secured.

## Background

Pakistan is the sixth most populous country in the world. With the second and third highest rates of stillbirths and newborn mortality [1], respectively, its progress towards achieving the targets specified in Millennium Development Goals 4 and 5 has been slow. Despite pioneering the launch of a family planning programme in the 1960s [2], Pakistan has a poor record in the reproductive health management of its own population. Nevertheless, the Pakistani health community deserves credit for its role in the reduction of the global burden of reproductive, maternal, newborn, and child health (RMNCH).

**Figure 1:**
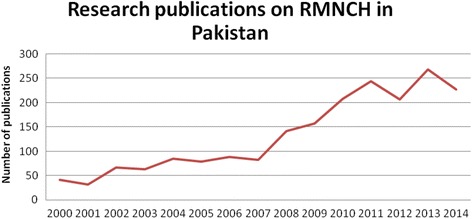
**Research publications on reproductive, maternal, newborn, and child health in Pakistan, 2000–2014.**

An adequate appreciation of Pakistan’s achievements and contributions requires that they be reviewed in context. Factors contributing to Pakistan’s poor progress in reducing RMNCH include its low level of female literacy, gender inequity, political challenges, and extremism with its associated relentless violence. Further, less than 1% of Pakistan’s GDP is allocated to the health sector [3]. Its expenditure record is certainly not outstanding, even compared to neighbouring countries with relatively poorer economic indicators, but it is reasonable given the challenges it continues to face. Notwithstanding, despite poor progress on the home front, the Pakistani health community, especially its RMNCH researchers, has contributed significantly to the global knowledge and evidence base in this field (Figure 1).

Unfortunately, these achievements have not been widely recognized, neither by national policy and decision-makers nor by the global health community at large. Exceptions include recognition by Richard Horton, editor of the *Lancet* and a keen observer of global knowledge power, through his acknowledgment of Pakistan’s contribution: “*Pakistan is a phenomenally research-productive nation, which has delivered important innovations in health services, and has produced leaders in health who have made a demonstrable difference both domestically and internationally*” [4]. To further prove this point, the present commentary provides selected examples of studies conducted and published by Pakistani researchers in the field of RMNCH, which have not only contributed to global knowledge, but have also been used as primary sources of evidence to formulate WHO guidelines in this area.

### Pakistani contributions to the field of reproductive, maternal, newborn, and child health (RMNCH)

In the late 1980s, Pakistan was part of an 11-country study on the aetiology of childhood pneumonia [5,6], which contributed to the development of the WHO standard pneumonia treatment guidelines [7] and the *Haemophilus influenzae B* and pneumococcal vaccines. Pioneering work by Pakistan’s National Institute of Health and National Acute Respiratory Infection Control Programme in the 1990s [8] led to examination of the relationship between *in vitro* antimicrobial resistance and *in vivo* outcomes for clinical pneumonia as children responded clinically to oral co-trimoxazole [9]. A randomised controlled trial (RCT) showed that co-trimoxazole and amoxicillin were equally effective in treating pneumonia associated with fast breathing in hospitalized children and that children with high *in vitro* antimicrobial resistance were cured with oral co-trimoxazole [10]. An additional finding that amoxicillin was twice as effective as co-trimoxazole for pneumonia with chest in-drawing was very important at a time when WHO guidelines recommended all children with chest in-drawing pneumonia be hospitalized for injectable antibiotic therapy [11]. A RCT in eight low- and middle-income countries [12] and in Pakistan [13] showed that oral amoxicillin was as effective as injectable penicillin/ampicillin for pneumonia with chest in-drawing in children aged 2–59 months. These results had a far-reaching impact in 2012 when WHO revised pneumonia treatment guidelines, recommending that oral amoxicillin be used to treat pneumonia with chest in-drawing on an outpatient basis in low-HIV settings [14]. Data from Pakistan also contributed to the revision of WHO guidelines [15] for the management of children with wheeze [16] and for shortening antibiotic treatment for fast-breathing pneumonia from 5 to 3 days [17] in order to reduce the scope for the development of antimicrobial resistance.

In reproductive health, Pakistani researchers have been central to many ground-breaking international studies which have influenced the design and implementation of reproductive health programmes. The most important among these is the Antenatal Corticosteroid Treatment trial from the National Institute of Child Health and Human Development group [18], which has served as a primary source informing the development of new, as yet unpublished WHO guidelines on the use of antenatal corticosteroids for the prevention of pre-term births. Cognizant of the scarcity of ultrasonography equipment in remote rural areas, Pakistani researchers have initiated studies to compare the accuracy of gestational age estimates obtained through ultrasonography with those obtained through history and physical examination [19]. They have also been pioneers in the study of mental health issues among pregnant women, evident from the work of Karmaliani et al. from Hyderabad and Sind [20,21], as well as forming part of large multi-country studies, such as that by Bloch et al. [22], to examine tobacco use and second-hand smoke prevalence among pregnant women.

Pakistan has also been a global leader in community-based strategies to expand access to RMNCH services. Learning from its lady health worker (LHW) programme, many countries have adopted integrated community-case management of common childhood illnesses as a strategy [23]. Additionally, with appropriate training, supervision, and regular supplies, LHWs have managed pneumonia with chest in-drawing in children aged 2–59 months at the community level in two cluster RCTs with low treatment failure rates and mortality [24,25]; these findings are the first of their kind globally and have stimulated further research (Personal Communication, Shamim Qazi, WHO HQ, Geneva, Switzerland). LHWs have also contributed to the reduction of perinatal mortality through the provision of services in the community [26], which has contributed to the development of WHO recommendations for home visits in the first week of life [15].

## Conclusions

Pakistani researchers have been able to contribute to RMNCH-related global knowledge and evidence base in the face of challenging conditions, and they should feel proud of these achievements despite the fact that the current state of RMNCH affairs in Pakistan does not offer exciting or satisfying prospects. Pakistan needs to invest in human and health-related resources to generate and use policy-relevant knowledge for its own population. In order for Pakistan to reduce the national burden of RMNCH it must be able to apply research evidence for improved policymaking. Therefore, it is essential that appropriate funding, fair incentives for research production, appropriate recognition by the government, improved capacities for policy- and decision-makers to demand and use evidence, and strong support from the development community are all urgently required.

## Abbreviations

LHW: Lady health worker
RCT: Randomised controlled trial
RMNCH: Reproductive, maternal, newborn, and child health.
